# Isolation and whole genomic analysis of mesophilic bacterium *Pseudoglutamicibacter cumminsii* in epithelial mesothelioma

**DOI:** 10.1016/j.heliyon.2024.e35617

**Published:** 2024-08-02

**Authors:** Nan Xu, Kunyi Wu, Ting La, Bo Cao

**Affiliations:** a Department of Clinical Laboratory, The Second Affiliated Hospital of Xi'an Jiaotong University, Xi'an, 710004, Shaanxi, China; b Core Research Laboratory, The Second Affiliated Hospital of Xi'an Jiaotong University, Xi'an, 710004, Shaanxi, China; c National-Local Joint Engineering Research Center of Biodiagnosis & Biotherapy, The Second Affiliated Hospital of Xi'an Jiaotong University, Xi'an, 710004, China

**Keywords:** *Pseudoglutamicibacter cumminsii*, Epithelial mesothelioma, Whole genome sequencing, Blood, Tumor, Metabolism

## Abstract

The relationship between bacteria and tumors has been the hot spot of clinical research in recent years. *Pseudoglutamicibacter cumminsii* is an aerobic Gram-positive bacterium commonly found in soil. Recent studies have identified *P. cumminsii* in patients with cutaneous and urinary tract infections. However, little is known on its pathogenesis as well as involvement in other clinical symptoms. In this study, we first report the isolation of *P. cumminsii* in blood of an epithelial mesothelioma patient. The clinical and laboratory characteristics of *P. cumminsii* were first described and evaluated. The pure colony of *P. cumminsii* was then identified using automated microorganism identification system and mass spectrum. The whole genome of the newly identified strain was sequenced with third generation sequencing (TGS). The assembled genome was further annotated and analyzed. Whole genomic and comparative genomic analysis revealed that the isolated *P. cumminsii* strain in this study had a genome size of 2,179,930 bp and had considerable unique genes compared with strains reported in previous findings. Further phylogenetic analysis showed that this strain had divergent phylogenetic relationship with other *P. cumminsii* strains. Based on these results, the newly found *P. cumminsii* strain was named *P. cumminsii* XJ001 (PC1). Virulence analysis identified a total of 71 pathogenic genes with potential roles in adherence, immune modulation, nutrition/metabolism, and regulation in PC1. Functional analysis demonstrated that the annotated genes in PC1 were mainly clustered into amino acid metabolism (168 genes), carbohydrate metabolism (107 genes), cofactor and vitamin metabolisms (98 genes), and energy metabolism (68 genes). Specifically, six genes including *yodJ*, *idh*, *katA*, *pyk*, *sodA*, and *glsA* were identified within cancer pathways, and their corresponding homologous genes have been documented with precise roles in human cancer. Collectively, the above results first identified *P. cumminsii* in the blood of tumor patients and further provide whole genomic landscape of the newly identified PC1 strain, shedding light on future studies of bacteria in tumorigenesis.

## Graphical abstract

**Figure:**
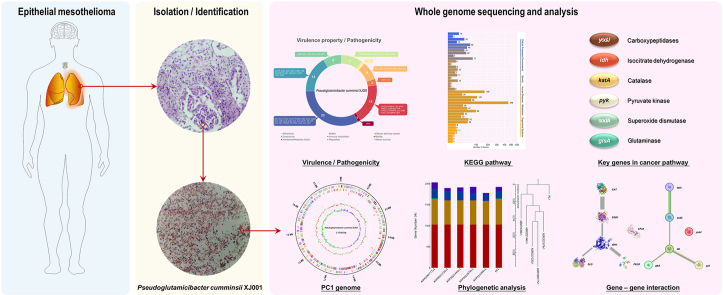

## 1 Introduction

The relationship between bacteria and tumors has been one of the hot topics of basic and clinical researches [1]. A well-known example is the finding of *Helicobacter pylori*. Previous studies showed direct role of *H. pylori* in the progression of gastric cancer [2]. In recent years, a series of studies have provided more evidences on the involvement of bacteria during tumorigenesis [3]. For instance, *Fusobacterium nucleatum* in colorectal cancer [4], *Porphyromonas gingivalis* in oral cancer [5], and intratumor microbiota in breast cancer [6]. Investigating the roles of bacteria and the underlying mechanisms in tumor patients will shed new light on innovative approaches to cancer therapy.

Recent studies revealed that bacteria play critical roles in the occurrence, development, metastasis and anti-chemotherapy of tumors [7,8]. Specifically, it is demonstrated that bacteria can promote tumor via multiple mechanisms including changing tumor microenvironment, generating proinflammatory microenvironment to stimulate cancer cell proliferation, and up-regulating the expression of oncogenic factors [7]. Nevertheless, little is reported for studies linking whole genome of bacteria and progression of tumor. Revealing the mechanisms in the view of genomics will be of significance for developing novel approaches for the prevention and treatment of cancer.

Epithelial mesothelioma is a rare and aggressive form of cancer that primarily affects the lining of the lungs, abdomen, or heart [9]. It is primarily caused by exposure to asbestos fibers, which can lead to the development of tumors in the mesothelial cells that make up these linings. Up to date, epithelial mesothelioma accounts for the majority of mesothelioma cases, with its distinct histological characteristics and clinical features [10]. However, the molecular mechanism of epithelial mesothelioma remains unknown.

*Pseudoglutamicibacter cumminsii* is an aerobic, catalase-positive, Gram-positive bacterium commonly found in soil [11]. Previous studies have identified *P. cumminsii* in patients with cutaneous and urinary tract infections. For instance, recently Puca et al. identified *P. cumminsii* in an immunosuppressed patient with urinary tract infection [12]. Nevertheless, little is known on its pathogenesis as well as involvement in other clinical symptoms.

In this study, we first isolated *P. cumminsii* in the blood sample of a patient with epithelial mesothelioma. To investigate the role of *P. cumminsii* in tumorigenesis, we conducted whole genome sequencing (WGS) and analyzing of this newly identified strain. Furthermore, the correlation between *P. cumminsii* infection and epithelial mesothelioma was discussed. Collectively, the aims of this study are: 1). to isolate and identify the *P. cumminsii* in epithelial mesothelioma; and 2). to conduct whole genomic analysis of the newly isolated *P. cumminsii*.

## 2 Materials and methods

### 2.1 Patients

The patient is a 36-year-old male presented with fatigue, weight loss, lower abdominal pain, and elevated body temperature without any apparent cause. On May 1, 2018, he was admitted to the Second Affiliated Hospital of Xi'an Jiaotong University for treatment. Prior to admission, abdominal CT and abdominal ultrasound were performed, revealing abdominal effusion and a mixed mass in the lower abdomen. Subsequently, biopsy puncture of peritoneal tumor was conducted, confirming the diagnosis of epithelial mesothelioma. The patient received chemotherapy with pemetrexed plus carboplatin for two cycles. The blood cell analysis showed WBC of 42.89 × 10^9^/L and N% of 91.90 %, with a high body temperature peaking at 39.5 °C. After 8 days of treatment with intravenous meropenem sodium/sulbactam sodium for infection, a recheck of blood cell analysis showed WBC of 26.97 × 10^9^/L and N% of 90.10 %. The treatment was then changed to intravenous imipenem/cilastatin sodium for anti-infection therapy. Simultaneously, two blood samples were cultured on August 2 and August 15, 2018, respectively.

### 2.2 Bacterial isolation, identification, and drug sensitivity testing

Blood culture bottles were incubated in an automated blood culture system. Once an alarm was triggered, the sample was immediately subcultured onto blood agar plates. After 24 h of incubation at 37 °C in an incubator, a single bacterial colony was selected for Gram staining and was observed under a light microscope. The colony was then identified using Merieux VITEK 2 Compact and Merieux MALDI-TOF MS mass spectrometry. For drug sensitivity testing, the interpretation reference for *Enterobacteriaceae* breakpoints in the European Committee on Antimicrobial Susceptibility Testing (EUCAST) 2021 guidelines was followed.

### 2.3 Genomic DNA extraction

*P. cumminsii* isolated from the patient's blood sample was cultured in LB broth at 30 °C and shaken at 200 rpm for 12 h. The bacterial cells were then collected by centrifugation at 8000 rpm for 2 min. Genomic DNA was extracted using the bacterial genomic DNA extraction kit from Tiangen Biotech. The quality of the DNA was assessed using agarose gel electrophoresis, and the concentration and purity of the samples were determined by measuring the OD value at 280 nm wavelength. Subsequently, the DNA samples were sent to BGI Biotech for sequencing analysis.

### 2.4 Genome sequencing and assembly

The *P. cumminsii* strain XJ001 (PC1) genome was sequenced using a third-generation Nanopore sequencing platform at the Beijing Genomics Institute (BGI, Shenzhen, China). Raw reads generated by the Nanopore platform were filtered by Porechop (version v0.2.4). Nanopore subreads (length <2 kb) were removed. The program Canu (version v1.5) was used for selfcorrection. Draft genomic unitigs, which are uncontested groups of fragments, were assembled using Canu. To improve the accuracy of genome sequences, GATK (version v3.4-0-g7e26428; https://www.broadinstitute.org/gatk/) was used to make single-base corrections. The whole genome sequence of PC1 was submitted to NCBI SRA database with BioProject ID of PRJNA1090324.

### 2.5 Genome component prediction

Gene prediction was performed on the *P. cumminsii* genome assembly by Glimmer3 (version v3.02; http://www.cbcb.umd.edu/software/glimmer/) with Hidden Markov models, which is a gene prediction tool that is particularly good at identifying coding regions in microbial genomes. Recognition of tRNA, rRNA and sRNAs was generated using tRNAscan-SE (version v1.3.1), RNAmmer (version v1.2), and Rfam (version v9.1), respectively. The tandem repeats annotation was obtained using the Tandem Repeat Finder (version v4.04; http://tandem.bu.edu/trf/trf.html), and the minisatellite DNA and microsatellite DNA were selected based on the number and length of repeat units. The CRISPR identification was predicted using CRISPRFinder (version v 4.2.20).

### 2.6 Comparative genomics and phylogenetic analysis

The genomes of five *P. cumminsii* strains including ASM1690777v1, ASM3021230v1, ASM3021440v1, ASM3021679v1 and ASM314399v1 reported in previous studies were used as references. The synteny of PC1 and reference strains was performed using MUMmer and BLAST. Core/Pan genes of PC1 and reference strains were clustered by the CD-HIT rapid clustering of similar proteins software with a threshold of 50 % pairwise identity and 0.7 length difference cutoff in amino acid. Gene family is constructed using multi software including BLAST (aligning the protein sequence), SOLAR (eliminating the redundancy) and Hcluster_sg (clustering gene family for the alignment results). The phylogenetic tree is constructed by the TreeBeST using the method of NJ.

### 2.7 Gene annotation and functional analysis

The best hit was abstracted using Blast alignment tool for function annotation. Six databases including InterPro Database (IPR), COG (Clusters of Orthologous Groups), GO (Gene Ontology), KEGG (Kyoto Encyclopedia of Genes and Genomes), SWISSPROT, and NR (Non-Redundant Protein Database databases), which are widely used to enrich and classify the high-level functions and utilities of microbial system (e.g., pathways and diseases), are used for general function annotation. Resistance genes and carbohydrates were identified based on the core dataset in ARDB (Antibiotic Resistance Genes Database) and CAZy (Carbohydrate-Active enZYmes Database), which are specifically designed to identify and analyze antibiotic resistance and carbohydrate-active genes, respectively. Type III secretion system effector proteins (T3SS) were detected in the EffectiveT3 database.

### 2.8 Virulence factor analysis

The virulence factor analysis for PC1 was performed utilizing the VFDB (Virulence Factor Database), an extensive and integrated online platform that specializes in the curation of data on virulence factors associated with bacterial pathogens [13]. The VFanalyzer tool, a component of the VFDB, was employed to identify known and potential virulence factors within the PC1 genome. Subsequently, these identified factors underwent further annotation and interpretation to elucidate their potential roles and pathogenicity.

### 2.9 Gene-gene interaction analysis

The gene-gene interaction analysis of key genes in PC1, which are annotated in cancer pathways, was further explored using the STRING database. This database is renowned for its focus on both known and predicted protein-protein interactions, offering a comprehensive view of interactions within a single species as well as across various organisms [14]. Homologous genes in human corresponding to the key genes identified in PC1 were utilized to construct a network of gene-gene interactions within the STRING database. Following this, the interactions observed in PC1 were predicted based on the patterns of gene-gene interactions identified in human.

### 2.10 Ethics

This study was approved by the Medical Ethics Committee of the Second Affiliated Hospital of Xi'an Jiaotong University (No. 2022240). Written informed consent was obtained from the patient for the publication of this study. Comprehensive voluntary participation, data security and anonymization were guaranteed through the study process according to ethical guidelines for clinical research.

## 3 Results

### 3.1 Clinical characteristics of patients

**Fig. 1:**
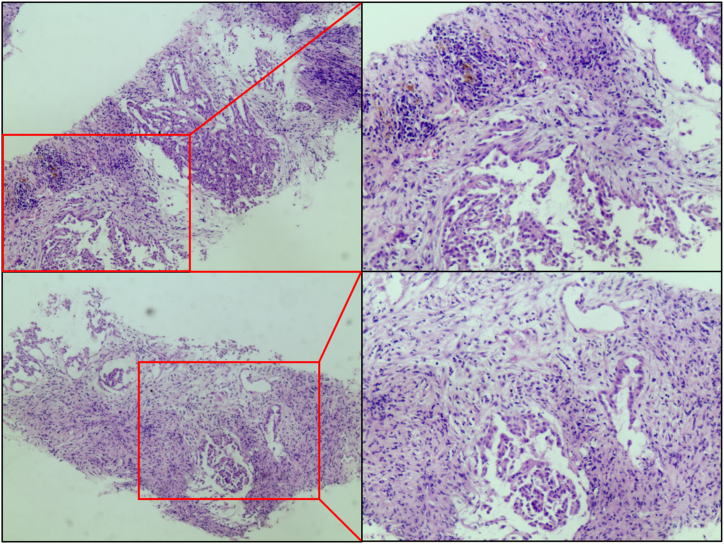
The pathological staining of patient with *P. cumminsii* infection. The cell boundaries of tumor cells were clearly defined, with rich and eosinophilic cytoplasm. The nuclei exhibited mild pleomorphism, and the nucleoli were prominently visible. Mitotic figures were rarely observed. In some areas, irregular solid, tubular-papillary, beam, and adenomatous structures were present, along with pleomorphic and lymphohistiocytoid cell morphology. Interstitial fibrosis was also noted.

Upon admission on May 1, 2018, the patient presented with a body temperature of 36.7 °C, a pulse rate of 94 beats per minute, a respiratory rate of 20 breaths per minute, a blood pressure of 102/61 mmHg, and clear consciousness. From May 2nd to May 7th, the patient received anti-infection therapy and fluid infusion. On May 9th, in consultation with the Respiratory Disease Department, the anti-infection therapy was modified to include imipenem in combination with vancomycin, resulting in a reduction in symptoms (Table 1). After six days, two blood samples were collected from peripheral veins and cultured, with positive results obtained after 24 h and 33 min for the first sample, and 20 h and 29 min for the second sample. At the same time, percutaneous peritoneal mass puncture was performed under ultrasound guidance, and the procedure was successfully completed. The tissue obtained from the peritoneal puncture was subjected to immunohistological analysis, which revealed CK(+) (cytokeratin), VIM(−) (vimentin), EMA(+) (epithelial membrane antigen), D2-40 small focus (+), CR(+) (calretinin), CK5/6(+), P53(+), CK-L(+), TTF(−) (thyroid transcription factor), CDX2(−), PSA(−) (prostate-specific antigen), and KI67(+) 20 %. The histological examination indicated the presence of an adenocarcinoma focus in the fibrous tissue of the peritoneal puncture and infiltration of hyperplastic tumor tissue. Based on the histological characteristics and immune markers, the diagnosis of epithelial mesothelioma was made (Fig. 1). Subsequently, an abdominal PET-CT scan was performed, revealing multiple lesions in the peritoneal cavity and pelvic cavity. The peritoneum and omentum were predominantly affected, with multiple enlarged lymph nodes exhibiting increased radiotracer uptake. The scan also indicated the presence of fluid retention in the chest and abdominal cavities, as well as encapsulation in the abdominal cavity and pelvic omentum space. These findings are consistent with the manifestation of abdominal malignant mesothelioma.

**Table 1:** Infection profile of patients after hospitalization.

Date	WBC (10^9^/L)	LYM (%)	NEU (%)	RBC (10^12^/L)	HGB (g)	PLT (10^9^/L)	CRP (mg/L)	PCT (ng/mL)	Body temperature (°C)
5.1	12.59	9.50	82.80	2.74	70	562	–	–	38
5.3	15.73	6.92	85.91	3.39	92	667	16.288	0.5–2.0	–
5.7	21.17	8.30	84.90	3.63	98	523	–	–	38
5.10	21.24	5.74	87.44	3.72	99	580	12.76	0.5	–
5.15	26.72	6.0	88.90	3.36	89	250	19.90	0.9	–
5.23	11.79	–	83.60	–	62	334	–	–	–

*Note*: The patient was admitted in hospital in 2018. Abbreviations for each item were: WBC - white blood cells; LYM - percentage of lymphocytes; NEU - percentage of neutrophils; RBC - red blood cells; HGB - hemoglobin; PLT - platelets; CRP - C-reactive protein; PCT - procalcitonin. “-” represents no data.

### 3.2 Laboratory characteristics of PC1

After obtaining a positive result from blood culturing, the isolated PC1 strain was cultured on a blood culture plate under 5 % CO_2_ at 37 °C for 24 h. Visual observation revealed a gray, moist, smooth, and opaque colony with an irregular border, measuring 2–3 mm in diameter. No hemolysis ring or distinct odor was detected (Figure s1). Gram staining exhibited the presence of Gram-positive bacilli, although some Gram-negative bacilli were also observed. Initially, the species could not be identified using Meropenem VITEK 2 Compact, but it was later confirmed to be *P. cumminsii* through mass spectrometry analysis. The minimum inhibitory concentration (MIC) values of the isolated strain for various drugs were determined as follows: linezolid 0.25 mg/L, levofloxacin 0.008 mg/L, ciprofloxacin 0.5 mg/L, imipenem 0.0048 mg/L, tetracycline 0.38 mg/L, vancomycin 0.25 mg/L, penicillin 0.024 mg/L, erythromycin 0.064 mg/L, gentamicin 0.5 mg/L, and clindamycin 0.024 mg/L. The isolated single PC1 strain was subsequently preserved for further research purposes.

### 3.3 Genome component analysis of PC1

**Fig. 2:**
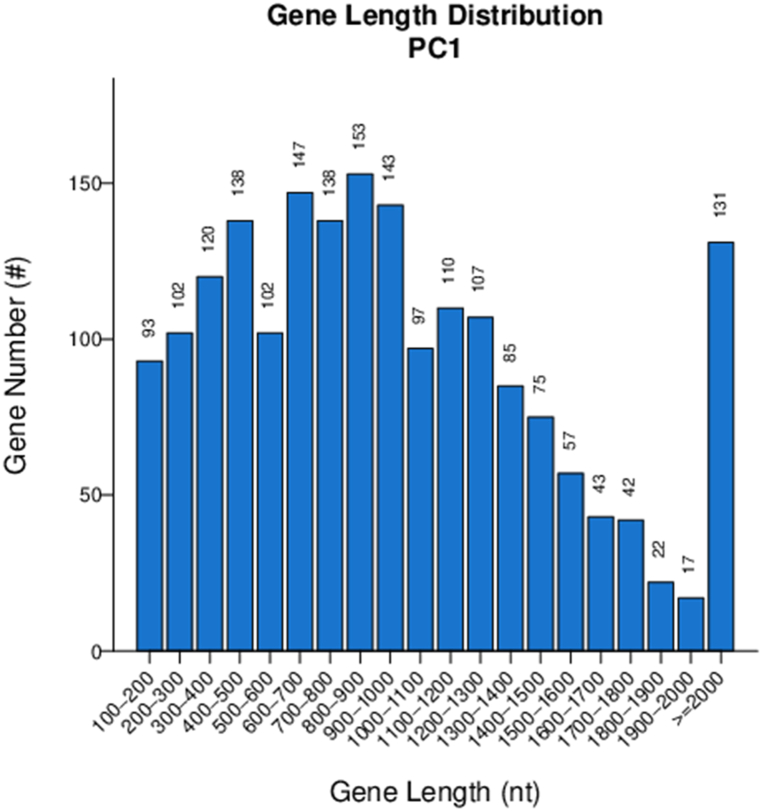
Gene length distribution of PC1 genome.

**Fig. 3:**
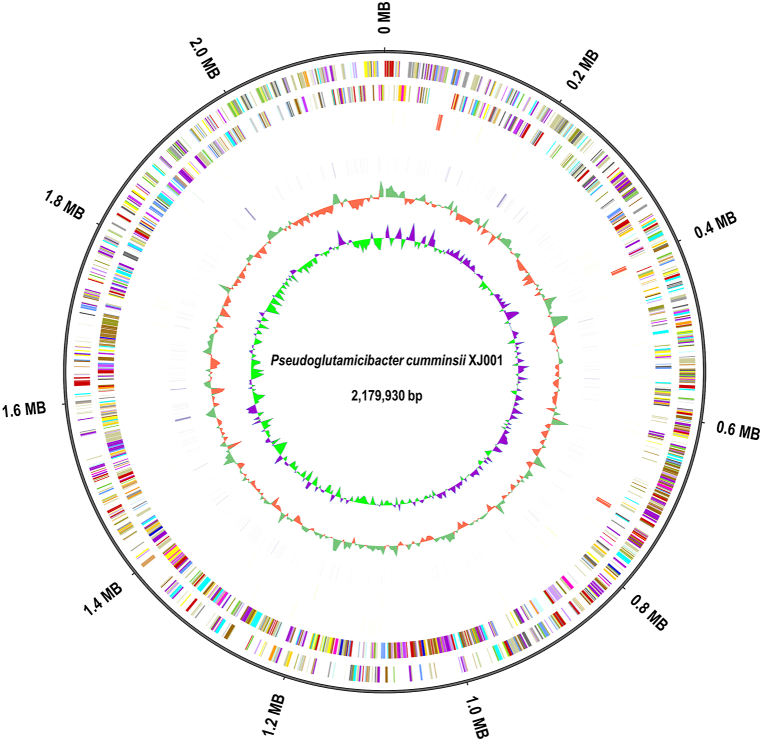
Circular representation of PC1 genome. From outer to inner: 1 - Genome Size; 2- Forward Strand Gene, colored according to cluster of orthologous groups (COG) classification; 3 - Reverse Strand Gene, colored according to cluster of orthologous groups (COG) classification; 4 - Forward Strand ncRNA; 5 - Reverse Strand ncRNA; 6 - repeat; 7 - GC; 8 - GC-SKEW.

The whole genome sequence of PC1 was found to have a size of 2,179,930 bp (Fig. 2). The total annotated genome length accounted for 1,922,406 bp and consisted of 1922 genes, with an average length of 1000.21 bp. The GC content of the genome was determined to be 61.23 %. The total gene length represented 88.19 % of the genome length (Fig. 3). Notably, PC1 exhibited the presence of several non-coding RNAs, including 47 tRNAs (constituting 0.16 % of the genome), three 5s_rRNAs (0.02 %), three 16s_rRNAs (0.21 %), three 23s_rRNAs (0.43 %), and three sRNAs (0.01 %). In terms of tandem repeats (TRs), PC1 displayed 332 TRs with repeat sizes ranging from 6 to 1305 bp, accounting for 1.04 % of the genome. The genome also contained 275 minisatellite DNA sequences (repeating in sizes of 15–61 bp, representing 0.56 % of the genome) and five microsatellite DNA sequences (repeating in sizes of 6–9 bp, accounting for 0.01 % of the genome). Additionally, PC1 harbored two potential CRISPR regions: one located from position 294,225 to 294,310 on the chromosome (85 bp), and the second region spanning from position 2,091,138 bp to 2,094,276 bp (3138 bp).

### 3.4 Comparative genomics of PC1

**Fig. 4:**
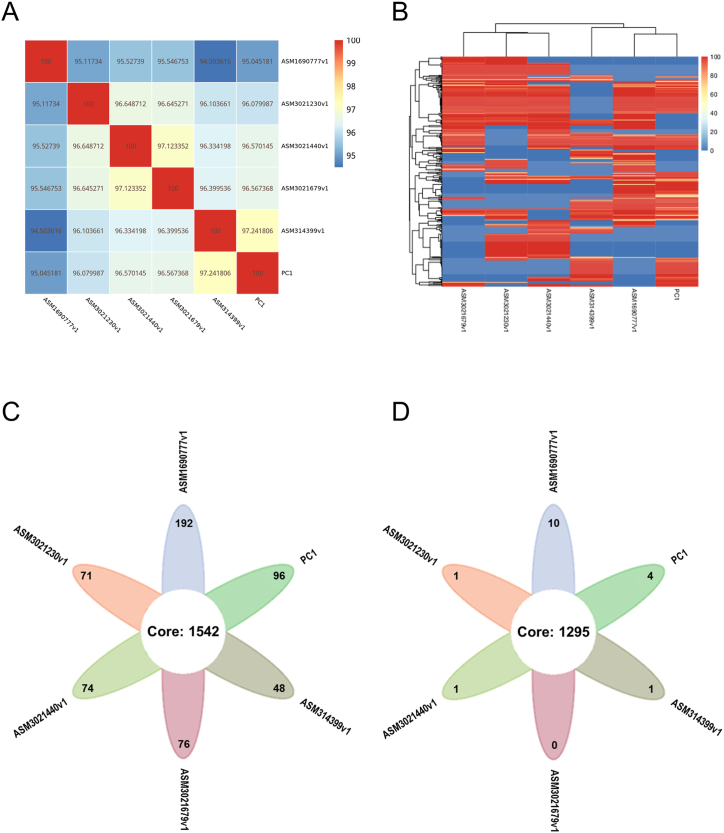
Comparative genomics of PC1 and other *P. cumminsii* strains. (A) ANI heatmap between strains. (B) Dispensable gene heat map. (C) Venn graph of pan genes. (D) Venn graph of orthologs in different gene family.

The structural variation of PC1 was compared with reference genomes from five strains, namely ASM1690777v1, ASM3021230v1, ASM3021440v1, ASM3021679v1, and ASM314399v1, as reported in previous studies (refer to Table 2 for detailed information on these strains). Results revealed that PC1 exhibited distinct structural variations compared to the other five strains (Figures s2-s7). The variation rates of nucleic acids and amino acids, when compared with ASM1690777v1, were 93.113 % and 97.73 % respectively. Similarly, when compared with ASM3021230v1, the rates were 91.775 % and 98.25 %, and with ASM3021440v1, the rates were 92.119 % and 98.59 %. Furthermore, when compared with ASM3021679v1, the rates were 91.769 % and 98.48 %, and with ASM314399v1, the rates were 94.040 % and 98.87 %. ANI (average nucleotide identity) analysis showed high average nucleotide identity values ranging from 94.50 % to 97.24 % between PC1 and the other strains, indicating significant genome similarity (Fig. 4). Additionally, PC1 and the other strains shared 1542 core genes (totaling 549,233 bp) and 2474 pan genes (totaling 785,925 bp) (Fig. 4). Notably, PC1 possessed 96 unique genes, suggesting distinct functionalities compared to the other strains. Regarding gene families, the five strains shared 1295 ortholog families, while PC1 had four unique gene families (Fig. 4).

**Table 2:** Reference strains and sample source of *Pseudoglutamicibacter cumminsii*.

*Pseudoglutamicibacter cumminsii* strain	Source
ASM1690777v1	–
ASM3021230v1	Urine Bladder
ASM3021440v1	Urine Bladder
ASM3021679v1	Urine Bladder
ASM314399v1	Cell culture
PC1	Blood

*Note*: “-” represents no available information of specific sample source. PC1 - *Pseudoglutamicibacter cumminsii* XJ001.

### 3.5 Phylogenetic analysis of PC1

**Fig. 5:**
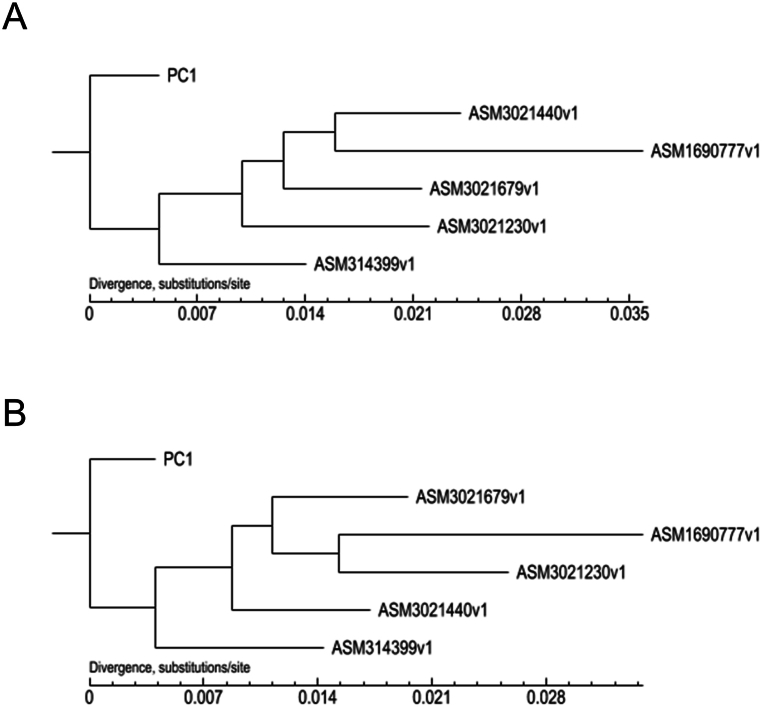
Phylogenetic tree of PC1 and other *P. cumminsii* strains. (A) Phylogenetic tree based on core/pan genes. (B) Phylogenetic tree based on gene family.

Phylogenetic trees were constructed based on core/pan genes and gene families to analyze the relationship between PC1 and the other five strains. The results revealed that PC1 exhibited divergence from the other strains in both core/pan gene tree and gene family tree (Fig. 5). This finding suggests that PC1, which was identified in blood sample from tumor patients, differs from the other strains isolated from other samples. Collectively, the above results may indicate its potentially distinct role in disease progression.

### 3.6 Overview of gene annotation analysis

Gene annotation analysis provides a comprehensive overview of the functional characteristics of PC1 genome. In general, a total of 1875 genes, accounting for 97.55 % of the genome, were annotated across various databases (Table 3; Tables s1-s3). The IPR database annotated 1626 genes (84.59 % of the genome), while SWISSPROT identified 844 genes (43.91 %). The COG database annotated 1409 genes (73.3 %), and the GO database classified 1254 genes (65.24 %) based on their molecular functions, biological processes, and cellular components. The KEGG database provided functional classifications for 1221 genes (63.52 %), while the NR annotated 1865 genes (97.03 %). The PC1 genome also showed the presence of virulence factors, with 85 genes (4.42 %) identified in the VFDB database. Additionally, antibiotic resistance genes were found in the ARDB database, with 5 genes (0.26 %) showing resistance to certain drugs. The CAZY database identified 25 genes (1.3 %) associated with carbohydrate metabolism. Moreover, the T3SS database annotated 473 genes (24.6 %) involved in the secretion of effector proteins. These annotations provide valuable insights into the genetic makeup and potential functions of the PC1 genome.

**Table 3:** Comparative analysis of PC1 genome annotation across diverse databases.

Database	Annotated number (%)
VFDB	85 (4.42 %)
ARDB	5 (0.26 %)
CAZY	25 (1.3 %)
IPR	1626 (84.59 %)
SWISSPROT	844 (43.91 %)
COG	1409 (73.3 %)
CARD	0 (0 %)
GO	1254 (65.24 %)
KEGG	1221 (63.52 %)
NR	1865 (97.03 %)
T3SS	473 (24.6 %)
Overall	1875 (97.55 %)

*Note*: Abbreviations for each item were: PC1 - *Pseudoglutamicibacter cumminsii* XJ001; VFDB - Virulence Factors of Pathogenic Bacteria Database; ARDB - Antibiotic Resistance Genes Database; CAZY - Carbohydrate-Active enZYmes Database; IPR - InterPro Database; COG - Cluster of Orthologous Groups of proteins; CARD - Comprehensive Antibiotic Resistance Database; GO - Gene Ontology; KEGG - Kyoto Encyclopedia of Genes and Genomes; NR - Non-Redundant Protein Database; T3SS - Type III Secretion System Effector Protein Database.

### 3.7 Virulence factor analysis

**Fig. 6:**
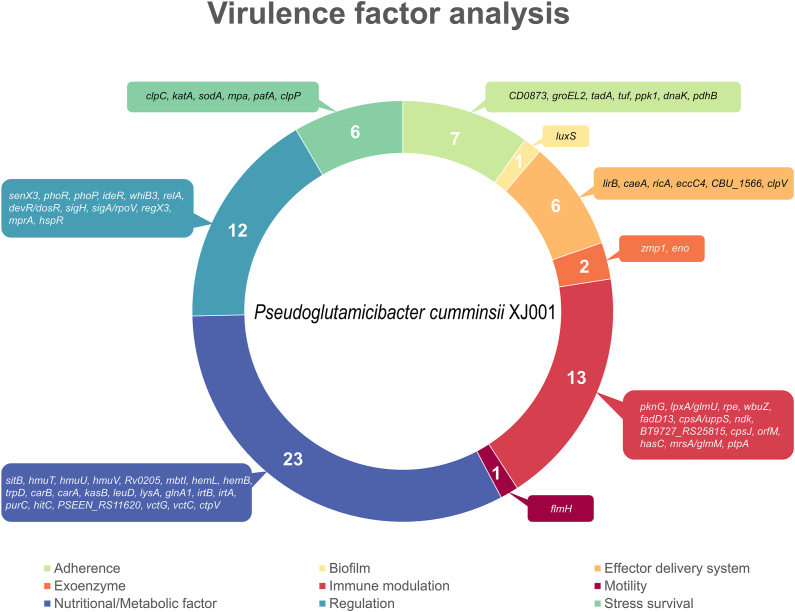
Virulence factor analysis of PC1 genome.

The virulence factor analysis identified a total of 71 potential virulence factors in PC1 (Fig. 6; Table s3). These virulence factors were further annotated in nine types, of which nutritional/metabolic factor had the most of genes (23 genes). The other main types mainly include immune modulation (13 genes), regulation (12 genes), adherence (7 genes), stress survival (6 genes), effector delivery system (6 genes), exoenzyme (2 genes), biofilm (1 gene), and motility (1 gene). Collectively, the identification of the above virulence factors will provide valuable references for elucidating the role of PC1 in tumorigenesis.

### 3.8 KEGG annotation analysis

**Fig. 7:**
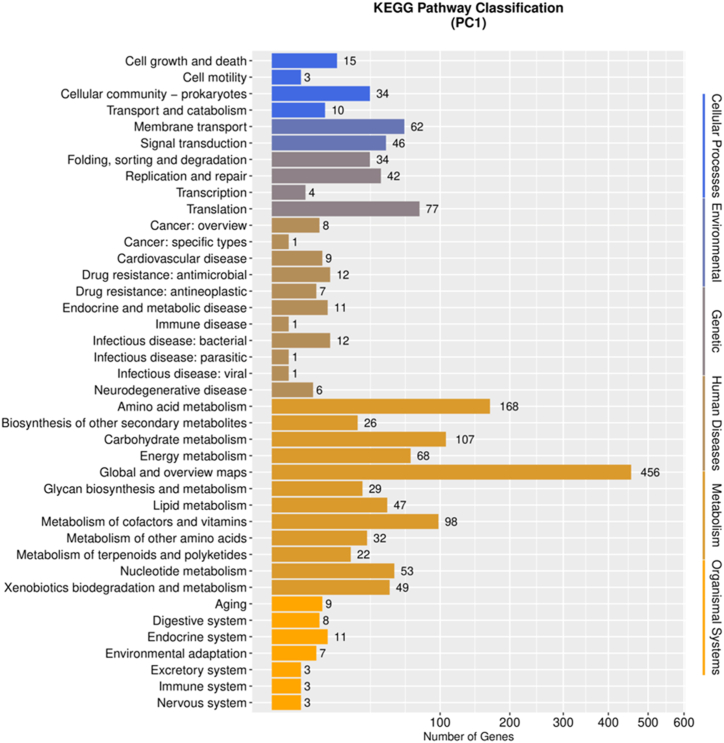
KEGG pathway classification of PC1 genome.

**Fig. 8:**
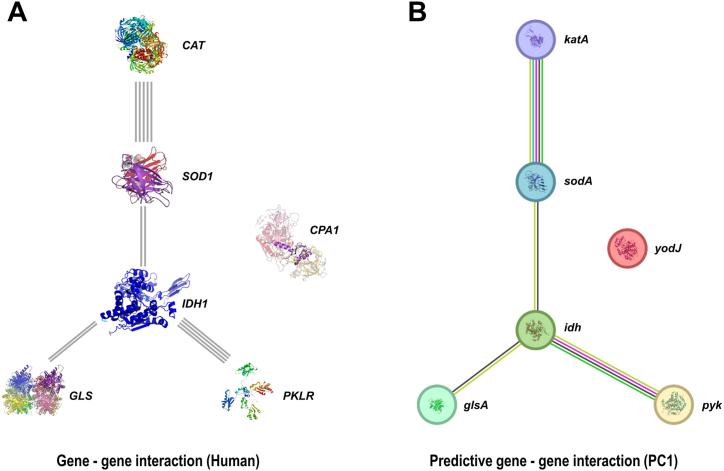
Gene-gene interaction analysis of key genes in cancer pathway in PC1 genome. (A) Homologous gene-gene interaction in human. (B) Predictive gene-gene interaction in PC1.

**Fig. 9:**
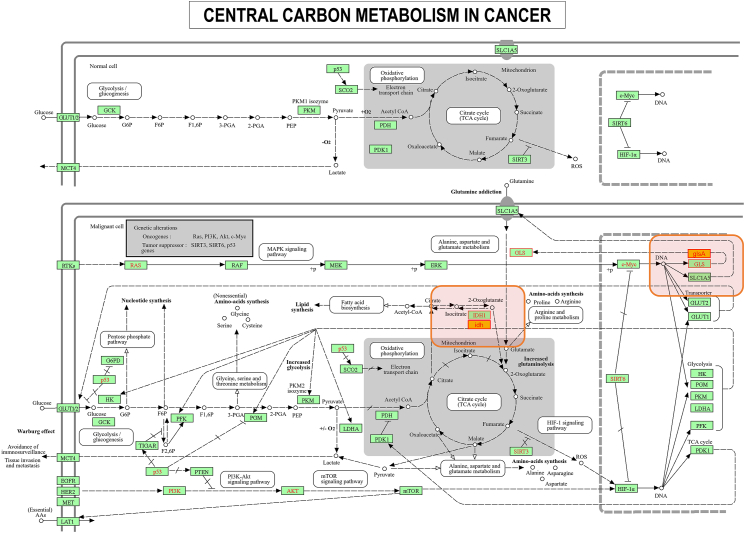
KEGG enrichment of key genes in cancer pathway in PC1 genome. Two key genes were enriched in the central carbon metabolism in cancer pathway. Highlighting box: green background with red letters - homologous genes in human; orange background with red letters - key genes in PC1.

In the KEGG classification, the PC1 strain exhibited variations in the number of genes across different categories (Fig. 7). The category of Metabolism had the highest number of genes, with 168 genes involved in amino acid metabolism, 107 genes in carbohydrate metabolism, 98 genes in the metabolism of cofactors and vitamins, 68 genes in energy metabolism, 53 genes in nucleotide metabolism, 49 genes in xenobiotic biodegradation and metabolism, and 47 genes in lipid metabolism. Under the category of Cellular processes, 34 genes were associated with cellular community-prokaryotes, 15 genes with cell growth and death, 10 genes with transport and catabolism, and 3 genes with cell motility. Notably, a considerable number of genes were found in the category of human diseases, including 12 genes related to infectious diseases caused by bacteria, 12 genes associated with antimicrobial drug resistance, and 11 genes involved in endocrine and metabolic diseases. Specifically, six genes were identified in the category of Cancer: overview, which included *yodJ*, *idh*, *katA*, *pyk*, *sodA*, and *glsA* (Table 4). Further gene-gene interaction analysis showed that the homologous genes of these genes in human had close interactions in metabolic pathways (Fig. 8). The five genes *CAT* (homologous gene *katA* in PC1; the same below), *SOD1* (*sodA*), *IDH1* (*idh*), GLS (*glsA*), and PKLR (*pyk*) showed distinct upstream and downstream expression pathways. Notably, two of the above key genes, *idh* and *glsA*, were enriched in the pathway of central carbon metabolism in cancer (Fig. 9). Taken together, these findings suggest that these metabolism-related key genes may play key roles in the progression of tumors in patients.

**Table 4:** Key genes in PC1 genome annotated in KEGG cancer pathway.

Gene (PC1)	Encoded protein	Function	Homologous gene (Human)	Reported role in cancer
*yodJ*	carboxypeptidase	catalyze the removal of amino acids from the C-terminus of proteins or peptides	*CPA*	[[21], [22], [23]]
*idh*	isocitrate dehydrogenase	catalyze a critical step in the citric acid cycle, converting isocitrate to alpha-ketoglutarate, while producing carbon dioxide and NADH	*IDH1*	[24,25]
*katA*	catalase	catalyze hydrogen peroxide into water and oxygen	*CAT*	[26,27]
*pyk*	pyruvate kinase	catalyze conversion of phosphoenolpyruvate to pyruvate, generating ATP	*PKLR*	[28,29]
*sodA*	superoxide dismutase	catalyze dismutation of superoxide radicals into hydrogen peroxide and molecular oxygen	*SOD1*	[30,31]
*glsA*	glutaminase	catalyze conversion of glutamine to glutamate	*GLS*	[32,33]

*Note*: PC1 - *Pseudoglutamicibacter cumminsii* XJ001.

### 3.9 GO annotation analysis

**Fig. 10:**
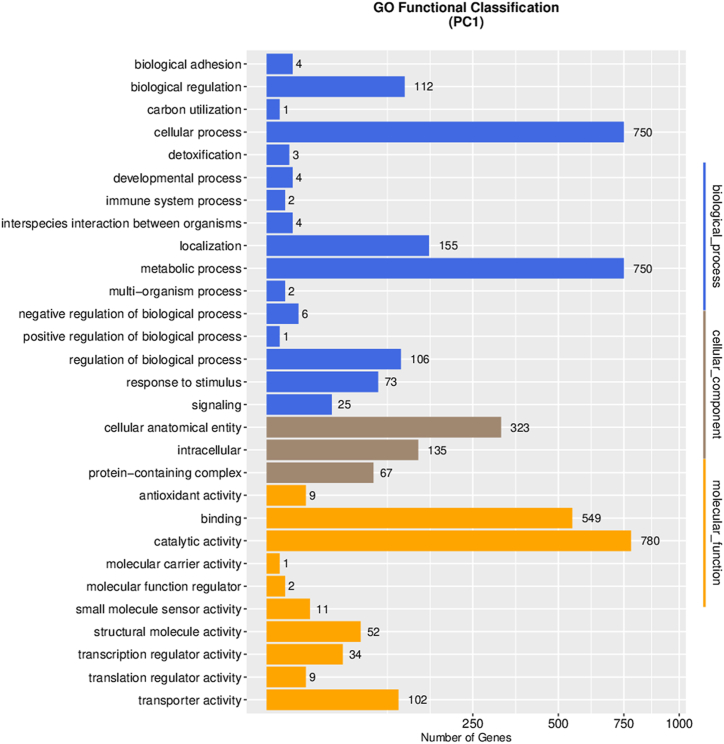
GO functional classification of PC1 genome.

In the GO classification, numerous genes were identified in different categories (Fig. 10). In the biological process category, there were 750 genes associated with cellular processes, 750 genes related to metabolic processes, 155 genes involved in localization, and 112 genes associated with biological regulation. In the cellular component category, there were 323 genes related to cellular anatomical entities, 135 genes associated with intracellular localization, and 67 genes associated with protein-containing complexes. In the molecular function category, there were 780 genes exhibiting catalytic activity, 549 genes involved in binding, and 102 genes with transporter activity. Collectively, the abundance of genes associated with metabolic processes underscores the significance of these pathways in cellular functioning and highlights their potential as targets for further investigation and therapeutic interventions.

### 3.10 COG annotation analysis

**Fig. 11:**
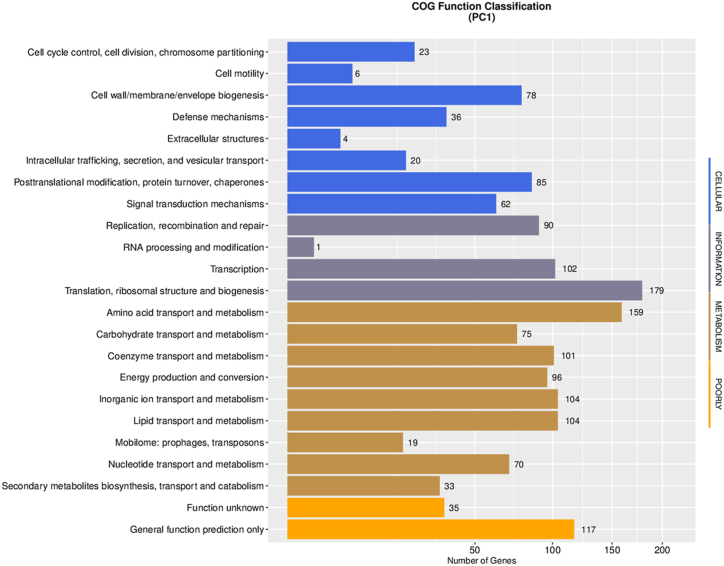
COG functional classification of PC1 genome.

In the COG classification, a detailed analysis revealed that the category of metabolism encompasses a substantial number of genes (Fig. 11). Among these, amino acid transport and metabolism accounted for the highest number of genes, with a total of 159 genes. Additionally, inorganic ion transport and metabolism, lipid transport and metabolism, coenzyme transport and metabolism, and energy production and conversion were also well-represented, with 104, 104, 101, and 96 genes, respectively. While in the cellular category, several important subcategories were identified. “Posttranslational modification, protein turnover, chaperones” stood out with 85 genes, indicating the crucial role of these processes in maintaining protein stability and cellular homeostasis. Additionally, “cell wall/membrane/envelope biogenesis” accounted for 78 genes, highlighting the importance of these processes in cellular structure and integrity. Moreover, “signal transduction mechanisms” were represented by 62 genes, suggesting their involvement in cellular communication and response. In the information category, the subcategory of “translation, ribosomal structure and biogenesis” exhibited the highest number of genes, with a total of 179 genes. Furthermore, the subcategories of “transcription” and “replication, recombination and repair” were represented by 102 and 90 genes, respectively, highlighting their significance in the regulation and maintenance of genetic information. Taken together, these results provide compelling evidence that metabolism is the primary category of genes within the COG classification, suggesting its pivotal role in various cellular processes. These findings further support the notion that metabolism is a vital component driving the genome function of PC1.

### 3.11 Annotation in other databases

In addition to gene annotations in KEGG, GO and COG classifications, there are also interesting annotations in other databases (Tables s1-s3). In the ARDB database, five genes have been identified with specific resistance properties. These include *trcR*, which confers resistance to vancomycin, *uppP*, which provides resistance against bacitracin, *mtrA*, which offers resistance to vancomycin and teicoplanin, *folA*, which imparts resistance to trimethoprim, and *yodJ*, which contributes to resistance against vancomycin. Furthermore, in the CAZy database, there are several genes related to carbohydrate processing. This includes three genes with Carbohydrate-Binding Modules (CBMs), two genes with Carbohydrate Esterases (CEs), nine genes with Glycoside Hydrolases (GHs), and ten genes with Glycosyl Transferases (GTs). These additional annotations from different databases provide further insights into the functional properties and roles of these genes in various cellular processes and antibiotic resistance mechanisms of PC1.

### 3.12 Clinical significance based on WGS results

Based on the above WGS results, the clinical significance of PC1 was further analyzed. For virulence and genomic annotation, WGS analysis revealed a series of pathogenic and tumor-related factors in PC1 genome (Fig. 6, Fig. 7, Fig. 8, Fig. 9). Correspondingly, the patient was concurrently treated with anti-infection measures alongside anti-tumor therapy. For antibiotic resistance, WGS analysis found resistance of PC1 to vancomycin, bacitracin, teicoplanin and trimethoprim, which was coincident with the result of MIC test (Table s1). Accordingly, the drugs of mezlocillin sodium and sulbactam sodium for injection and imipenem and cilastatin sodium for injection were used in further anti-biotic therapy. In addition, comparative genomics with other strains may offer significant insights for future clinical diagnostics (e.g., tumor-related genes in PC1).

## 4 Discussion

Bacteria have been found to play a significant role in promoting tumor growth through various mechanisms. One such mechanism involves the generation of a proinflammatory microenvironment that stimulates the proliferation of cancer cells. Bacterial infections can trigger an immune response, leading to the release of proinflammatory molecules that create a favorable environment for cancer cell growth and division [15]. Additionally, bacterial metabolites can induce changes in the tumor microenvironment, further supporting tumor progression. For example, some bacteria release metabolites that promote angiogenesis, the formation of new blood vessels, which enhances the nutrient supply to tumors and facilitates their growth and spread [16]. Moreover, bacteria can up-regulate the expression of oncogenic factors in host cells, contributing to the development of tumors. Through the secretion of virulence factors and toxins, bacteria can directly manipulate signaling pathways within host cells, leading to uncontrolled cell growth and division. By inducing DNA damage or activating specific oncogenes, bacteria disrupt the normal cellular balance, thus promoting the initiation and progression of cancer [17]. Furthermore, bacterial infections can result in immunosuppression, weakening the host's immune response against tumor cells. Bacteria have the ability to inhibit the function of immune cells, including T cells and natural killer cells, thereby allowing tumor cells to evade immune surveillance [18]. This immune evasion enables tumor cells to proliferate and metastasize more effectively. Understanding these mechanisms is crucial in developing strategies to target bacterial-induced tumor promotion and improve cancer treatment outcomes.

In this study, we first isolated and identified *P. cumminsii* in the blood of a patient with epithelial mesothelioma. However, the relationship between *P. cumminsii* infection and tumorigenesis remains unclear. One possible explanation is that the patient was previously infected by *P. cumminsii*, and continuous infection of *P. cumminsii* thus contributes to the initiation and progression of tumor. Another explanation is regarding tumor-caused immune suppression. When the patient's immune system is compromised, the invasive *P. cumminsii* can trigger bloodstream infections. In general, it is time-consuming and labor-intensive to elucidate the role and mechanism of *P. cumminsii* using conventional laboratory approaches.

Pathogenic microbial genomics offers a promising approach to tackle these issues effectively. Through whole genome sequencing and analysis, we discovered that the newly identified strain possesses a significant number of genes associated with virulence, antibiotic resistance, and bacterial metabolism. Specifically, a total of 71 pathogenic genes with potential roles in adherence, immune modulation, nutrition/metabolism, and regulation were identified as potential virulence factors (Fig. 6; Table s3). Previous studies have demonstrated that virulence factors of bacteria play pivotal roles in tumorigenesis. For instance, the CagA and VacA protein in *H. pylori* have been found directly responsible for the tumorigenesis of gastric cancer [19,20]. Correspondingly, PC1 may contribute to tumorigenesis through similar mechanism by virulence factors. Future studies should focus on the virulence property and pathogenicity of such virulence factors.

Notably, we identified several key genes (*yodJ*, *idh*, *katA*, *pyk*, *sodA*, and *glsA*) that are directly linked to bacterial metabolism. WGS analysis showed that the homologous genes of these genes in human had close gene-gene interactions in metabolic pathways, of which two genes involved in central carbon metabolism in cancer (Fig. 9). Given this, we propose that *P. cumminsii* may contribute to tumor progression by influencing metabolic pathways. The detailed roles of these key genes in human tumorigenesis were discussed as below.

Carboxypeptidases (encoded by *yodJ*) are enzymes that catalyze the removal of amino acids from the C-terminus of proteins or peptides. They play important roles in various physiological processes, including protein degradation, peptide signaling, and regulation of peptide hormone activity. Several studies have shown altered expression and activity levels of different carboxypeptidases in various types of tumors. For example, carboxypeptidase A1 (CPA1) has been found to be overexpressed in pancreatic cancer [21], while carboxypeptidase E (CPE) has been implicated in the progression of colorectal cancer [22]. In addition, CPE has been shown to be important in driving growth, survival and metastasis in many cancer types [23].

Isocitrate dehydrogenase (encoded by *idh*) is an enzyme that catalyzes a critical step in the citric acid cycle, converting isocitrate to alpha-ketoglutarate, while producing carbon dioxide and NADH. Mutations in the IDH1 and IDH2 genes are commonly found in certain types of tumors (particularly in brain tumors known as gliomas) and result in the production of an oncometabolite called 2-hydroxyglutarate (2-HG). The accumulation of 2-HG has been shown to have profound effects on cellular metabolism and epigenetic regulation. Elevated levels of 2-HG can inhibit various enzymes, including those involved in the regulation of DNA and histone methylation, leading to widespread changes in gene expression and cellular differentiation [24]. The IDH mutations have important implications for tumor development and progression. They are considered early genetic events in the pathogenesis of gliomas and are associated with younger patient age, better prognosis, and prolonged overall survival. IDH mutations have also been identified in other types of tumors, including acute myeloid leukemia (AML) and chondrosarcoma, but with different frequencies and clinical implications [25].

Catalase (encoded by *katA*) is an important enzyme that plays a crucial role in cellular antioxidant defense by breaking down hydrogen peroxide (H_2_O_2_) into water and oxygen. It is primarily located in peroxisomes, which are cellular organelles involved in various metabolic processes, including the metabolism of reactive oxygen species (ROS). excessive accumulation of ROS can lead to oxidative stress, DNA damage, and cellular dysfunction, which are associated with the development and progression of cancer. Increased catalase expression and activity in tumors may promote cancer cell survival and progression [26]. Higher levels of catalase can confer resistance to oxidative stress, protect cancer cells from ROS-induced damage, and promote cell survival and proliferation [27].

Pyruvate kinase (encoded by *pyk*) is an enzyme involved in the final step of glycolysis, which is the breakdown of glucose to produce energy in the form of ATP. PK catalyzes the conversion of phosphoenolpyruvate (PEP) to pyruvate, generating ATP in the process. The isoform of pyruvate kinase known as pyruvate kinase M2 (PKM2) has been extensively studied in the context of tumors. PKM2 is found to be highly expressed in many cancer cells, and it plays a role in promoting tumor growth and survival. PKM2 can undergo a specific form of regulation called “PKM2 dimerization,” which can influence its enzymatic activity and metabolic pathways [28]. PKM2 dimerization favors the diversion of glycolytic intermediates towards biosynthetic pathways that support tumor growth, such as nucleotide and lipid synthesis. In addition, recent findings demonstrated that PKM2 is associated with the Warburg effect and is thought to contribute to the metabolic changes observed in cancer cells [29].

Superoxide dismutase (encoded by *sodA*) is an enzyme that plays a crucial role in cellular antioxidant defense by catalyzing the dismutation of superoxide radicals into hydrogen peroxide and molecular oxygen. These ROS can cause damage to cellular components if not properly regulated. Production of ROS can also contribute to tumor development and progression. In some cases, increased levels of ROS can activate signaling pathways that promote cell growth, survival, and angiogenesis, all of which are important for tumor progressio). Inn ([30] this context, the role of SOD becomes more nuanced. While SOD helps regulate ROS levels, excessive or uncontrolled ROS scavenging by SOD might prevent the activation of signaling pathways that promote tumor suppression [31].

Glutaminase (encoded by *glsA*) is an enzyme that plays a critical role in cellular metabolism by catalyzing the conversion of glutamine to glutamate. Glutamine is an essential amino acid that serves as a major nutrient for cancer cells, especially in conditions where glucose availability is limited or compromised, such as during tumor growth. In many cancer types, including breast, lung, prostate, and brain cancers, there is evidence of increased glutaminase expression compared to normal tissues. This upregulation of glutaminase enables cancer cells to utilize glutamine as a fuel source and support their enhanced metabolism and growth [32]. Meanwhile, cancer cells often exhibit a phenomenon known as “glutamine addiction,” meaning they are highly dependent on glutamine for survival and growth. Glutaminase activity is essential for the production of glutamate, which can be further utilized in various metabolic pathways to support tumor growth [33]. Additionally, glutaminase-mediated production of glutamate can contribute to the generation of ROS, which can promote DNA damage and genomic instability, further facilitating tumor progression.

Furthermore, the above findings had potential clinical implications for cancer diagnostics and therapy. For instance, evaluating risk of cancer in patients infected with *P. cumminsii*, and developing specific therapy for *P. cumminsii* infection based on WGS results. Future large-scale observation and mechanism studies are needed to reveal the pathogenic role of *P. cumminsii* in cancer.

In conclusion, in this study we first isolated the new strain *P. cumminsii* XJ001 in the blood of a patient with epithelial mesothelioma. The clinical characteristics of the patient and laboratory characteristics of *P. cumminsii* were described and evaluated. The whole genome of this new strain was sequenced by TGS Nanopore sequencing. The phylogenetic, comparative and functional genomics of *P. cumminsii* were annotated and analyzed. Specifically, several key genes related to tumor progression were identified and discussed. Collectively, these results will contribute to our understanding of *P. cumminsii*'s role in cancer, particularly mesothelioma. The identified unique genes associated with cancer pathways will provide valuable references for diagnostic/therapeutic applications as well as future research on bacteria-tumor interactions. In addition, the limitation of this study is the lack of direct evidence linking *P. cumminsii* infection and tumor progression. A more thorough examination of the study's limitations in the future, especially regarding the lack of gene expression data, would provide a more balanced perspective. Further studies are needed to explore the role of *P. cumminsii* in other types of cancer, study its impact on the tumor microenvironment, and further characterize genetic markers to better understand its pathogenicity.

## Availability of data and materials

The data reported in this paper have been deposited in the NCBI SRA database (accession no. PRJNA1090324). The other data of whole genomic analysis were provided in the supplementary file.

## CRediT authorship contribution statement

**Nan Xu:** Writing – original draft, Validation, Resources, Investigation. **Kunyi Wu:** Visualization, Validation. **Ting La:** Writing – review & editing, Supervision. **Bo Cao:** Writing – review & editing, Writing – original draft, Supervision, Methodology, Conceptualization.

## Declaration of competing interest

The authors declare that they have no known competing financial interests or personal relationships that could have appeared to influence the work reported in this paper.
