# Supplementary material for: Isolation and whole genomic analysis of mesophilic bacterium Pseudoglutamicibacter cumminsii in epithelial mesothelioma
Source: Heliyon. 2024 Aug 2;10(15):e35617. doi: 10.1016/j.heliyon.2024.e35617 (PMC11336841; doi:10.1016/j.heliyon.2024.e35617)
Supplement: Multimedia component 1 [file mmc1.docx]

**Supporting information for:**

Isolation and whole genomic analysis of mesophilic bacterium *Pseudoglutamicibacter cumminsii* in epithelial mesothelioma

Nan Xu^1^, Kunyi Wu^2^, Ting La^3^, Bo Cao^1,2*^

^1^ Department of Clinical Laboratory, The Second Affiliated Hospital of Xi'an Jiaotong University, Xi’an 710004, Shaanxi, China

^2^ Core Research Laboratory, The Second Affiliated Hospital of Xi'an Jiaotong University, Xi’an 710004, Shaanxi, China

^3^ National-Local Joint Engineering Research Center of Biodiagnosis & Biotherapy, The Second Affiliated Hospital of Xi'an Jiaotong University, Xi'an 710004, China

Running title: Genomics of *P. cumminsii* in tumor

*Address for correspondence:

Bo Cao

Core Research Laboratory, The Second Affiliated Hospital of Xi'an Jiaotong University

157 West Fifth Road

Xi’an 710004, China

Tel: +86-159-9162-6728

Email: [bo_cao@xjtu.edu.cn](mailto:bo_cao@xjtu.edu.cn)

*For: Heliyon*


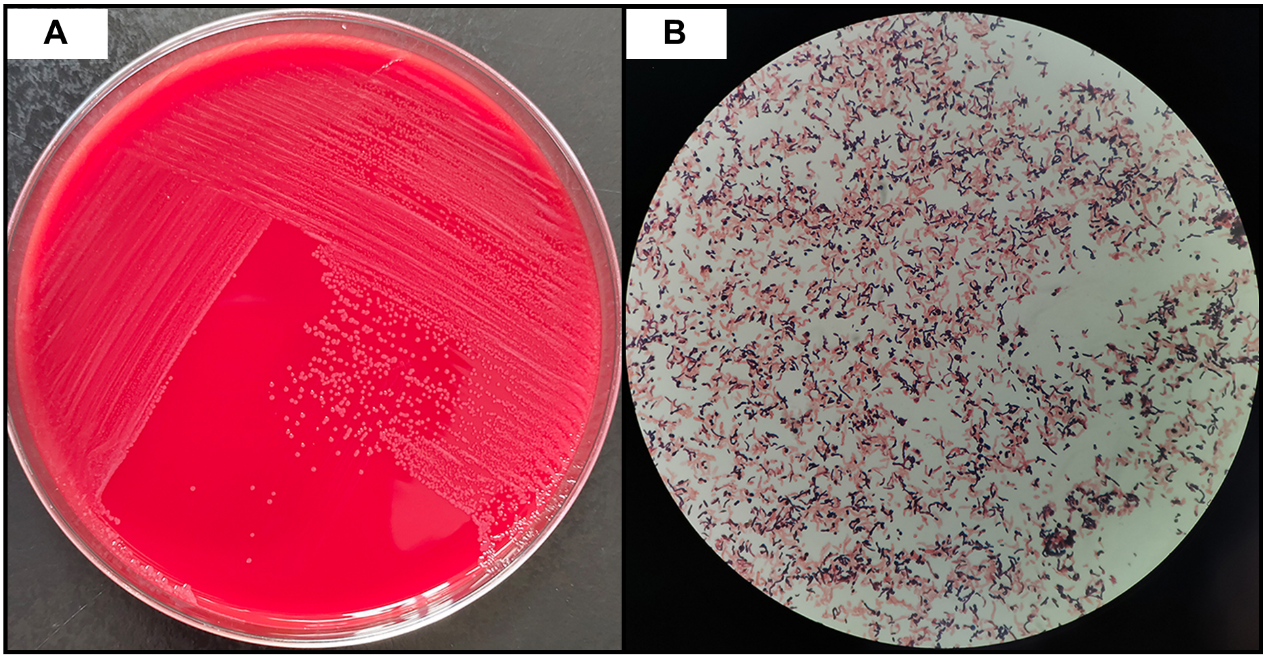


**Figure S1.** The morphology of isolated *Pseudoglutamicibacter cumminsii* in the blood of patient with epithelial mesothelioma.


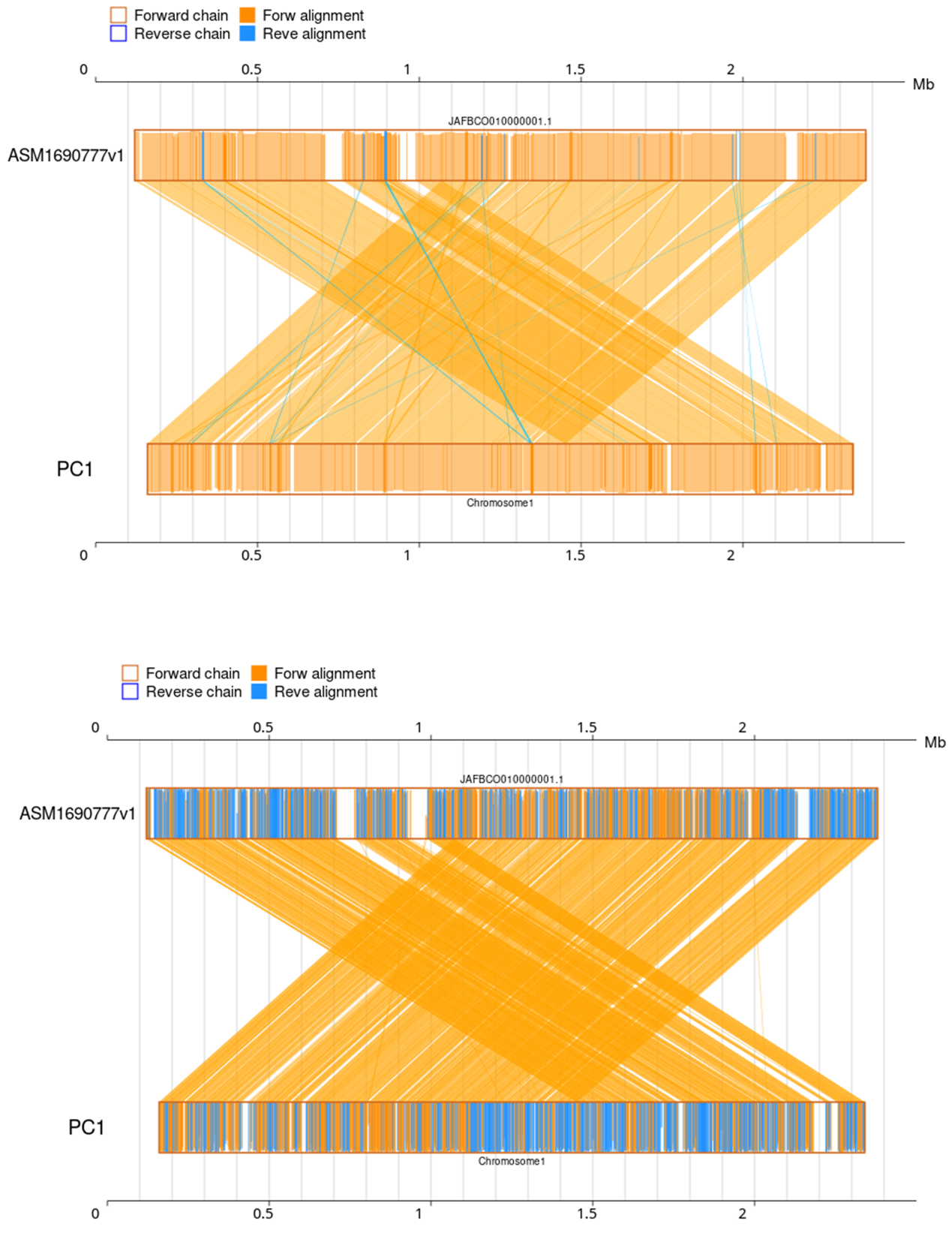


**Figure S2.** The nucleic acid level synteny and animo acid level synteny between *Pseudoglutamicibacter cumminsii* XJ001 strain and ASM1690777v1 strain.


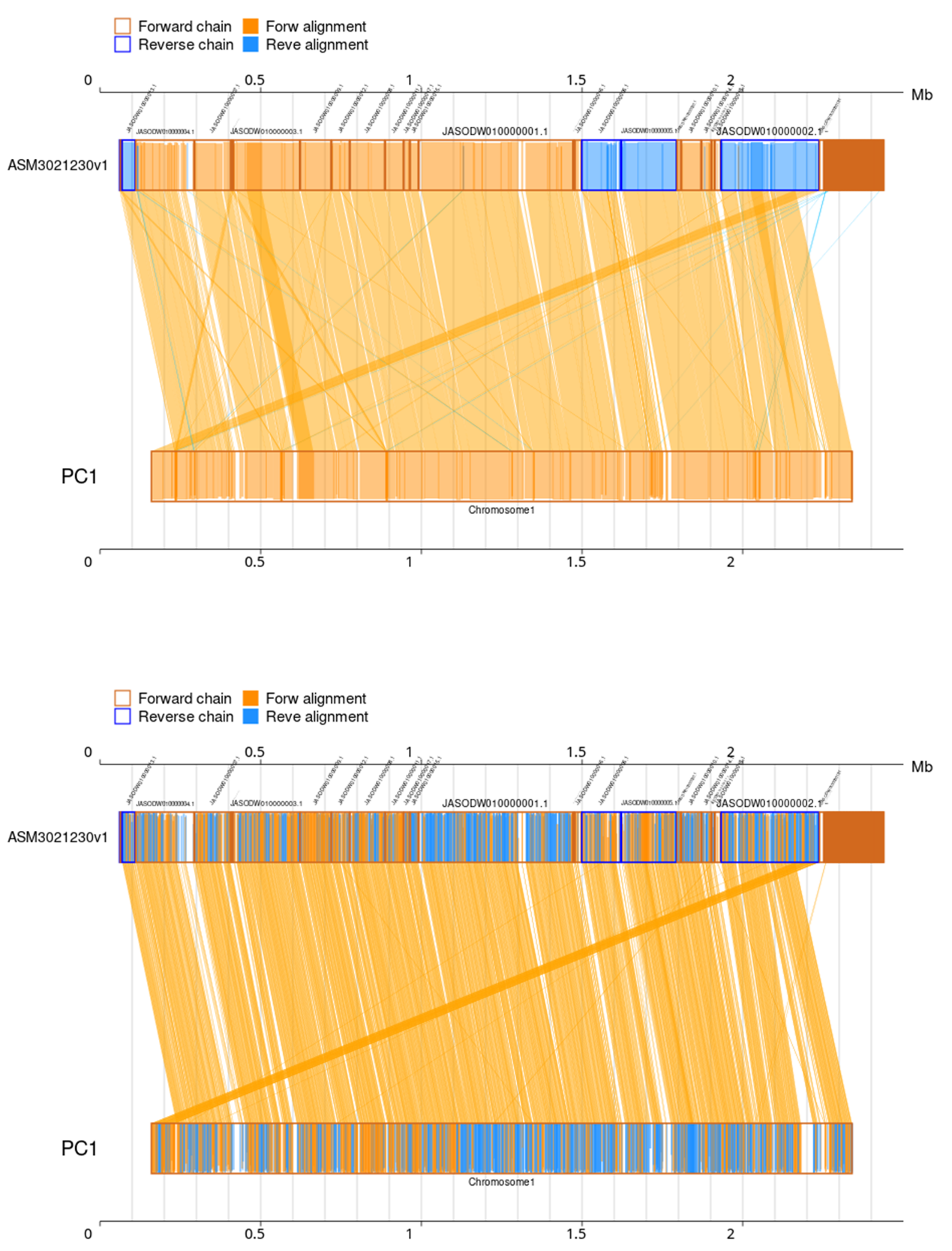


**Figure S3.** The nucleic acid level synteny and animo acid level synteny between *Pseudoglutamicibacter cumminsii* XJ001 strain and ASM3021230v1 strain.


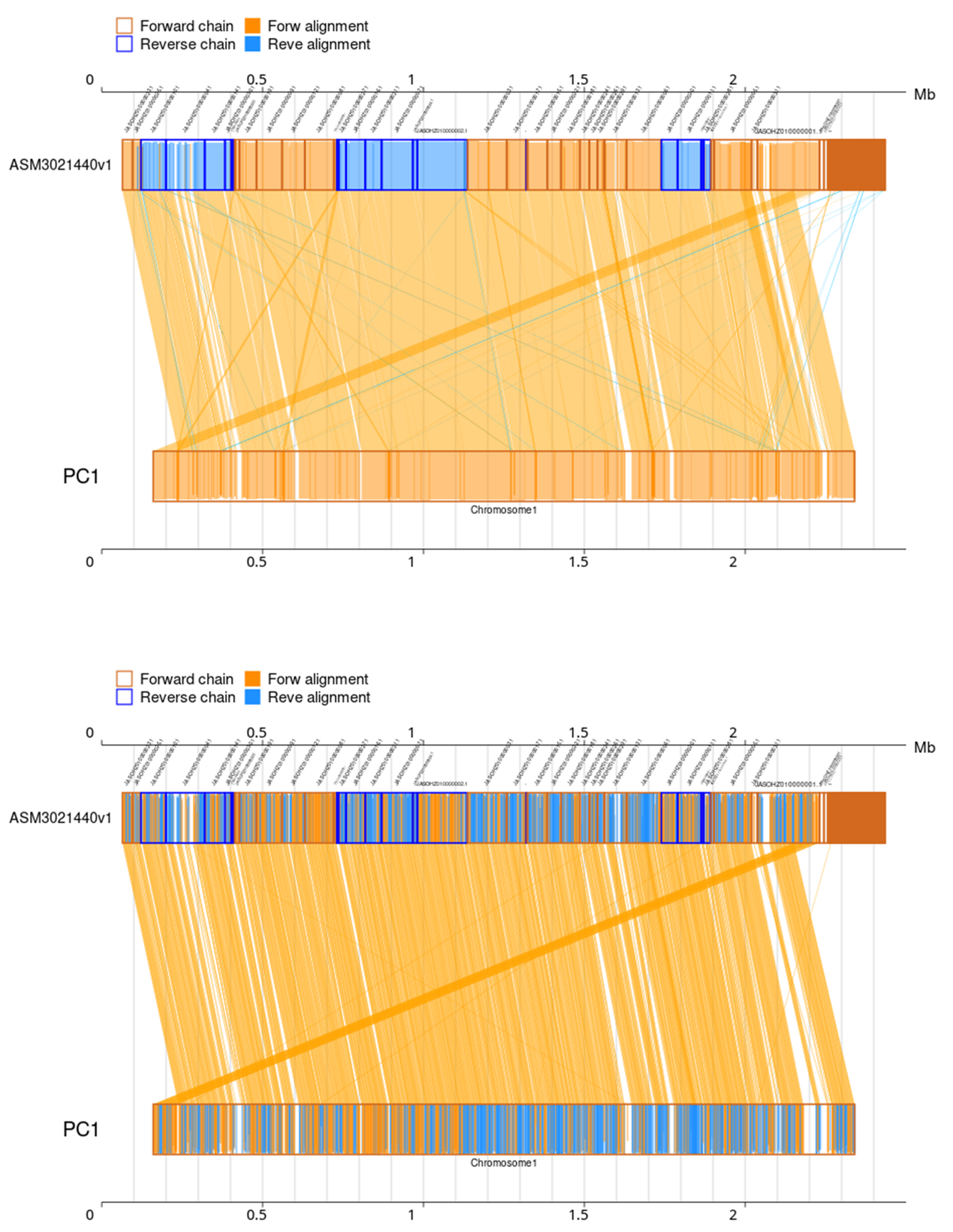


**Figure S4.** The nucleic acid level synteny and animo acid level synteny between *Pseudoglutamicibacter cumminsii* XJ001 strain and ASM3021440v1 strain.


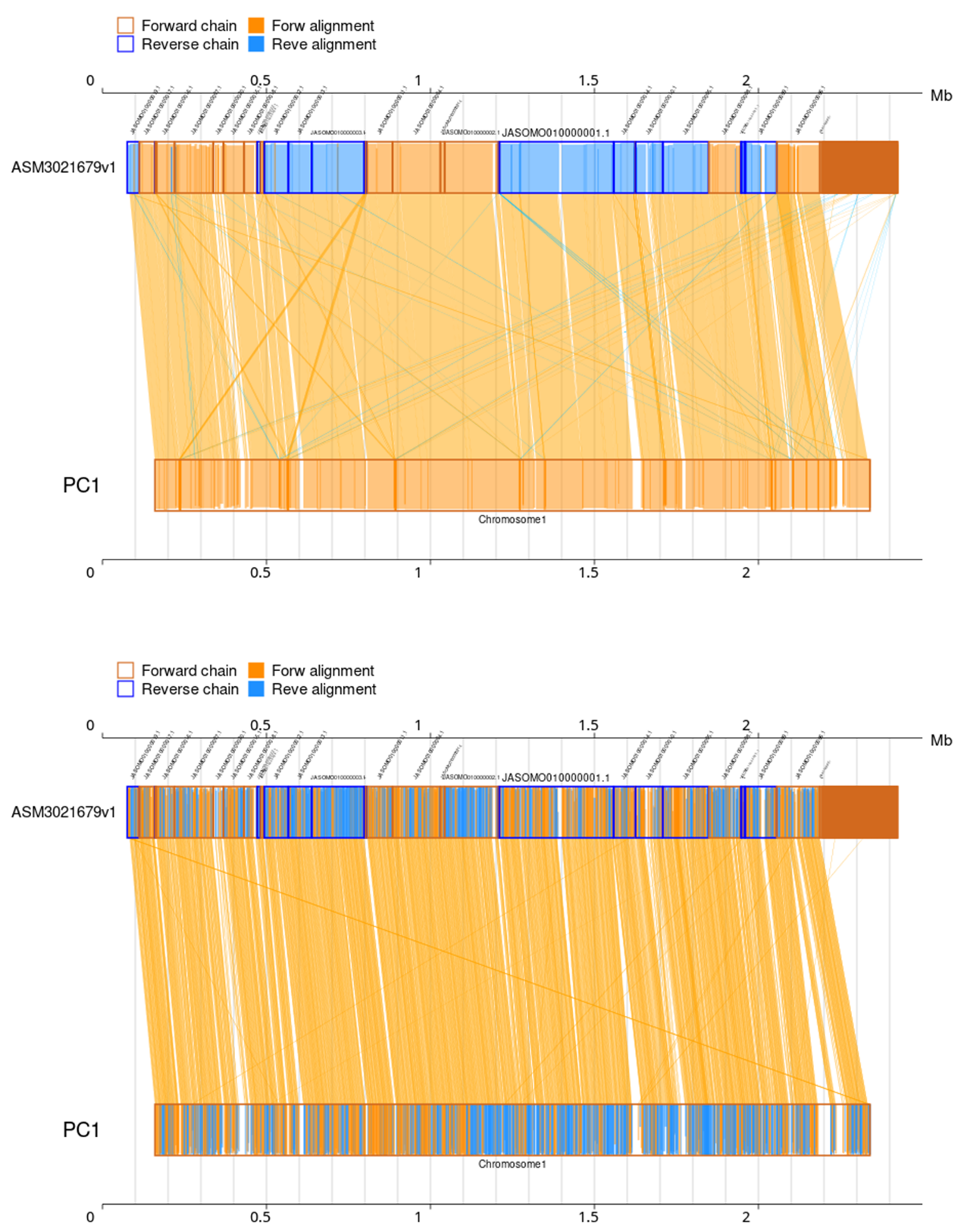


**Figure S5.** The nucleic acid level synteny and animo acid level synteny between *Pseudoglutamicibacter cumminsii* XJ001 strain and ASM3021679v1 strain.


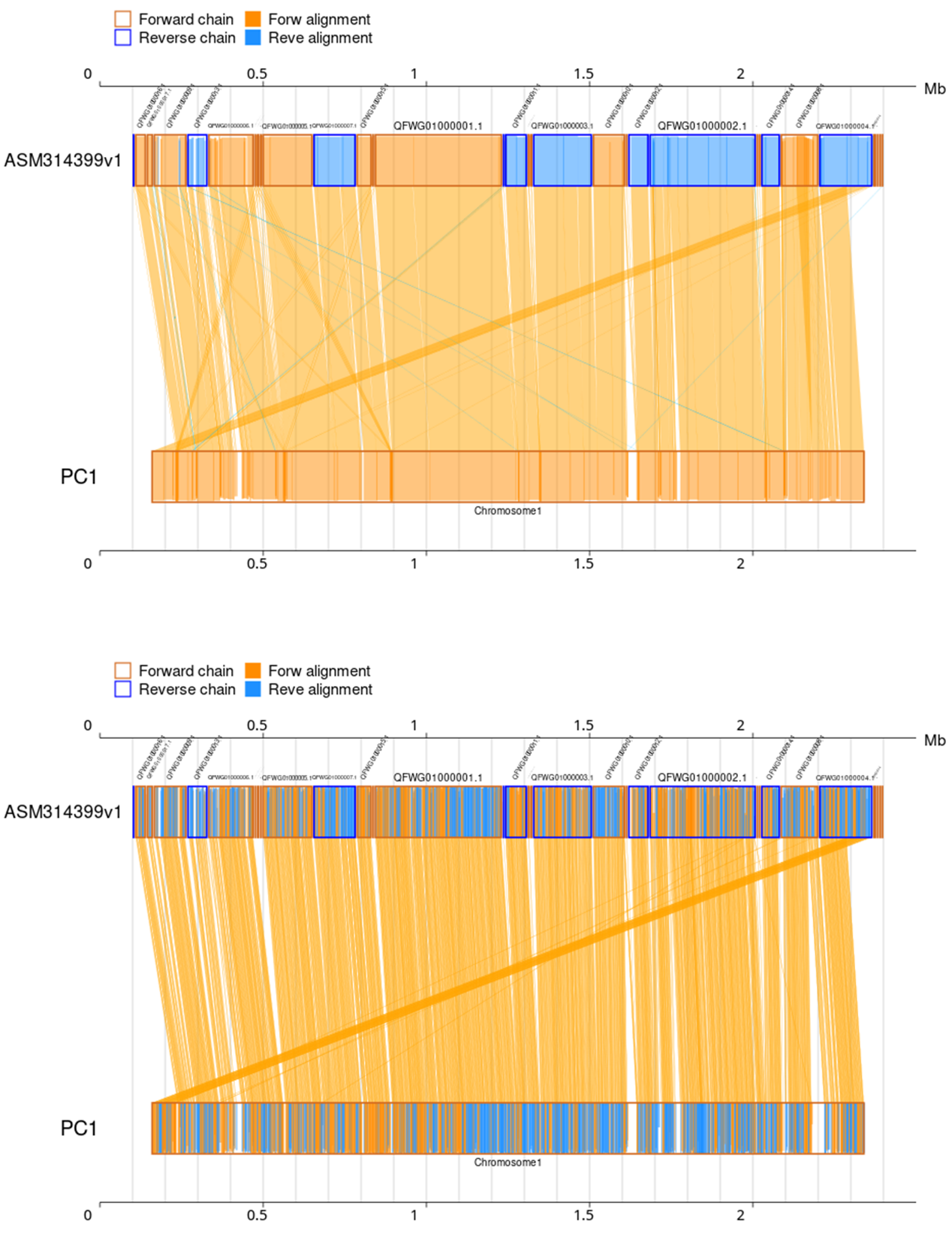


**Figure S6.** The nucleic acid level synteny and animo acid level synteny between *Pseudoglutamicibacter cumminsii* XJ001 strain and ASM314399v1 strain.


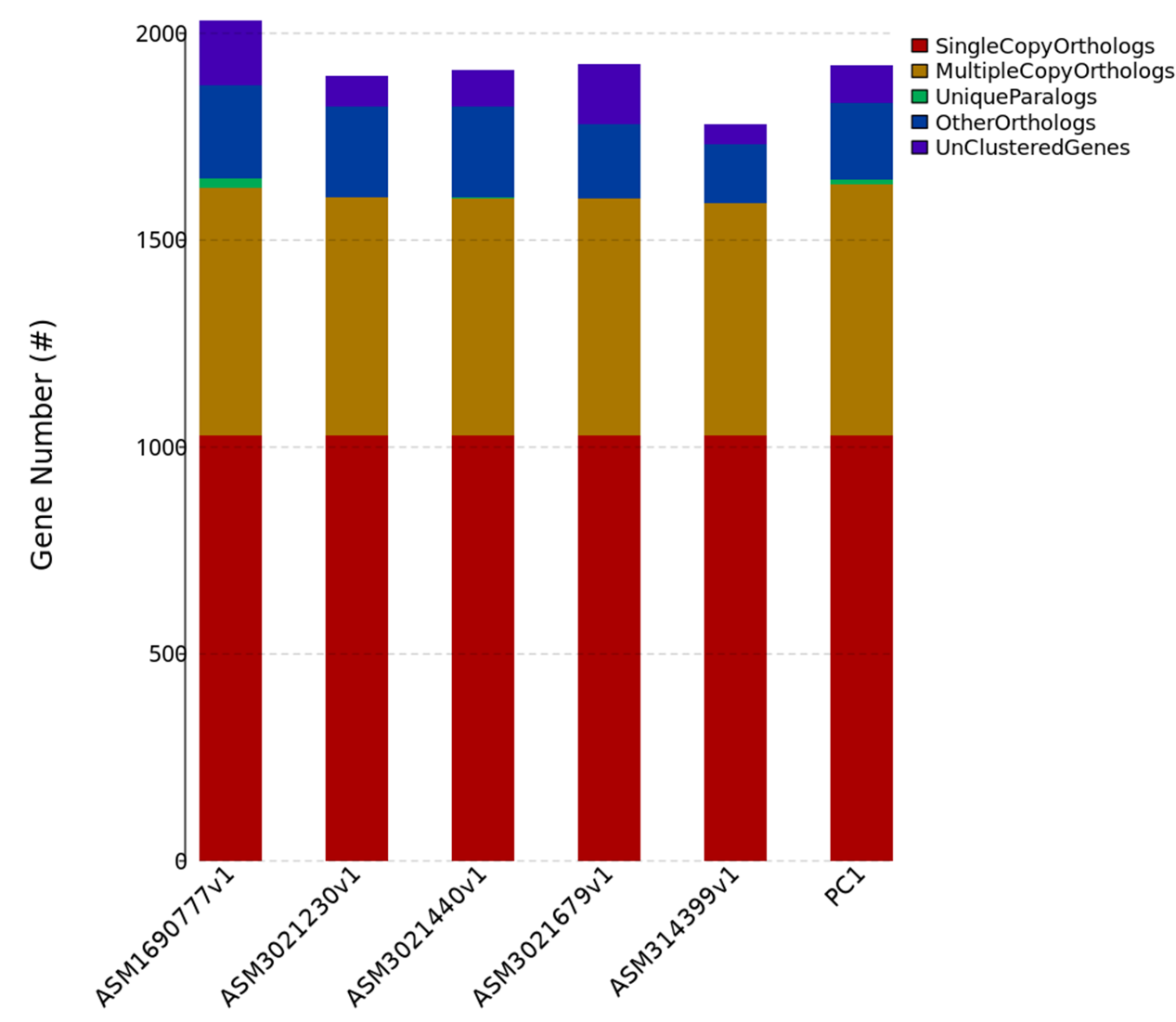


**Figure S7.** The number of orthologs between *Pseudoglutamicibacter cumminsii* XJ001 strain and other strains.
